# Longitudinal Analysis of Estrogen Receptor Gene Methylation, Estradiol, and Depressive Symptoms During the Perinatal Period

**DOI:** 10.1007/s12035-025-05556-3

**Published:** 2025-12-01

**Authors:** Gianna Zorzini, Alexandra Johann, Jelena Dukic, Elena Gardini, Ulrike Ehlert

**Affiliations:** Department of Clinical Psychology and Psychotherapy, University of Zurich, Binzmuehlestrasse 14/Box 26, 8050 Zurich, Switzerland

**Keywords:** Estrogen receptor, Epigenetics, DNA methylation, Perinatal depression

## Abstract

DNA methylation of estrogen receptor genes (*ESR1*, *ESR2*, and *GPER*) may affect the expression of the estrogen receptors (ERs) alpha, beta, and G protein-coupled estrogen receptor (GPER). Altered receptor expression may in turn affect the receptors’ sensitivity to estrogen, thereby modulating vulnerability to depression during periods of estrogen fluctuation. The aim of this study was to investigate the association between the methylation of *ESR1*, *ESR2*, and *GPER*, depressive symptoms, and estradiol during the perinatal period using a longitudinal design. A total of 159 women were followed longitudinally from 34 to 36 weeks of gestation to 8–12 weeks postpartum. Depressive symptoms were assessed using the Edinburgh Postnatal Depression Scale (EPDS). Salivary estradiol levels were quantified, and DNA methylation was analyzed using dried blood spots. Multivariate linear regressions and paired *t*-tests were used for analysis. Depressive symptoms were negatively associated with the mean overall *ESR1* methylation during pregnancy (*β* = −0.41*, p* = 0.002*)*, but not during the postpartum period (*p* ≥ 0.05). Haplotype CA during pregnancy (*p* ≤ 0.001) and haplotype TA (*p* = 0.03) during postpartum modified the relationship between depressive symptoms and overall mean *ESR1* DNAm. No associations emerged for *ESR2*, *GPER*, or estradiol at either time point (all *p* ≥ 0.05). The mean overall methylation of *ESR1* increased from pregnancy to postpartum (*t* = −2.59, *p* = 0.012) and was positively associated with depressive symptom scores during pregnancy (*β* = 0.418, *p* = 0.031). This work suggests that lower DNA methylation levels of *ESR1*, which may reflect higher ER-alpha expression and thus greater sensitivity to estrogen, are associated with increased depressive symptoms during pregnancy. The findings highlight molecular pathways of estrogen sensitivity, particularly via ER-alpha, as a promising target for future biomarker research for perinatal depression.

**Supplementary Information:**

The online version contains supplementary material available at 10.1007/s12035-025-05556-3.

## Background

Women are particularly susceptible to depression during phases of pronounced sex steroid hormone fluctuations, such as the premenstrual, perinatal, and perimenopausal periods [1–3]. Moreover, the occurrence of depression during one of these reproductive transitions appears to be associated with an increased risk of depression during the respective other phases [4–6], leading to the suggestion of a reproductive subtype of depression [7]. There is growing evidence to support the existence of this subtype, particularly regarding perinatal depression, which refers to depression arising during pregnancy or up to 1 year postpartum [8]. Depression during this period seems to be characterized by distinct symptoms, severity, heritability patterns, and epigenetic marks compared to depression occurring outside reproductive transitions [9–11]. Moreover, with a reported prevalence of 17%, and presumably even higher rates of undetected cases, the perinatal period represents a time of high vulnerability to developing depression [12, 13]. Perinatal depression is particularly concerning if left undetected and therefore untreated, as it is associated with far-reaching implications, not only for the woman but also for her offspring [14–16]. However, despite the well-documented prevalence, symptomatology, and consequences of perinatal depression, the biological underpinnings remain unclear, therefore, impeding the identification of reliable biomarkers for early detection and thus treatment initiation [17].

Although each female reproductive transition is characterized by unique fluctuations in sex steroid hormones, these fluctuations are considered important biological triggers for depression during reproductive transition phases [11]. Regarding the perinatal period, sex steroid hormones are found to increase strongly during pregnancy, followed by a sharp decrease after delivery [18]. However, only some women appear to be sensitive to these fluctuations and therefore vulnerable to depression during this period [19–21]. For instance, studies examining affective symptoms in multiparous women following a pharmacological simulation of the perinatal hormonal fluctuations have reported an increase in depressive symptoms in women with a history of perinatal depression but not in those without [22, 23]. Research exploring this sensitivity at the molecular level has specifically highlighted the involvement of estrogen signaling pathways. In detail, women with perinatal depression were found to exhibit an enrichment of transcripts involved in estrogen signaling compared to healthy controls, indicating an increased sensitivity to estrogen in affected women [24]. As such, sensitivity to estrogen has been suggested as a key pathway in mediating susceptibility to perinatal depression [21, 24, 25].

The physiological response to fluctuating estrogen levels depends on the capacity of estrogen receptors (ERs) [26]. The ERs, encompassing ER-α, ER-β, and the G protein-coupled ER (GPER), mediate the biological effects of estrogen through genomic and non-genomic pathways after binding to the hormone [27]. Given the widespread distribution of ERs throughout the body, the biological effects of estrogen are manifold [28]. In the brain, ERs are predominantly expressed in limbic regions, where estrogen has been found to modulate neurotransmitters such as gamma-aminobutyric acid (GABA), serotonin, dopamine, and glutamate, ultimately regulating emotional and cognitive functions [29–31]. However, the efficacy of the ERs in responding to fluctuating estrogen levels depends, at least in part, on their expression levels [32, 33]

DNA methylation (DNAm) of the ER genes *ESR1*, *ESR2*, and *GPER*, which encode ER-α, ER-β, and GPER, respectively, has the function of adjusting the expression levels of the receptors [34]. DNAm refers to the binding of methyl groups at cytosines in cytosine-guanine dinucleotides (CpGs), without changing the underlying DNA sequence itself [35]. This epigenetic modification can alter DNA accessibility, thereby affecting gene transcription and gene expression [36–38]. For instance, hypermethylation of the promoter regions of ER genes has been associated with reduced gene expression, while hypomethylation has been linked to increased ER levels [39–41].

Although several epigenome-wide association studies (EWAS) have identified associations between DNAm marks implicated in estrogen signaling and depression during the perinatal period, biomarkers for clinical use remain to be investigated [17, 25, 42, 43]. While EWAS offer broad insights across the whole genome, they are often burdened by multiple testing correction, which reduces the statistical power and thus the ability to detect subtle but biologically meaningful associations [44]. However, studies using a targeted approach to investigate ER gene methylation in association with perinatal depression are lacking [45]. Furthermore, several genetic variations in sex steroid receptor genes have been found to affect DNAm [38]. Previous work has also shown that specific genotypes modified the association between depression and DNAm [46, 47]. However, the potential role of ER gene variations in shaping ER gene DNAm and/or moderating the association between perinatal depressive symptoms and DNAm remains unexplored. Moreover, DNAm has the ability to change over time, which has also been observed during the perinatal period [48, 49]. Consequently, it is important to take this variability into consideration in order to identify reliable biomarkers [50]. While longitudinal courses of DNAm and potentially implicated factors remain understudied, findings from a cross-sectional study by our research group suggest that DNAm of ER genes may change over time [51]. In detail, differences in ESR1 DNAm levels emerged between pre- and postmenopausal women, and estradiol (E2) levels were found to be positively associated with *ESR1* DNAm in both groups. Thus, in view of the pronounced estrogen fluctuations during the perinatal period and the observed effects of estrogen on DNAm at various CpGs [18, 52], estrogen may play a crucial role in epigenetic regulation during the perinatal period. However, to date, no study has longitudinally examined DNAm of the ER genes, its relationship to estradiol, and its possible association with depressive symptoms during the perinatal period. This research gap limits the ability to form a comprehensive understanding and the identification of biomarkers for early detection.

The aim of this study was to investigate the association between DNAm of promoter regions in *ESR1*, *ESR2*, and *GPER*, depressive symptoms, and E2 levels during the perinatal period using a longitudinal design. Based on previous research and theoretical considerations, we hypothesized that depressive symptoms and E2 levels would be associated with DNAm levels of promoter regions in *ESR1*,* ESR2*, and *GPER* in pregnant and postpartum women. In addition, potential modifying effects of ER gene variations on the association between perinatal depressive symptoms and DNAm were investigated. We further assumed that these DNAm marks would change during the transition from pregnancy to the postpartum period and that this change would be associated with depressive symptoms and E2 levels.

## Methods

This study was part of a larger longitudinal research project on (epi-)genetic, biological, and psychological factors implicated in female mood disorders during the transition from pregnancy to postpartum. All participants provided informed consent prior to data collection, which took place between June 2019 and June 2021. The research project was carried out at the University of Zurich, Department of Clinical Psychology and Psychotherapy. The project was approved by the Ethics Committee of the Canton of Zurich (KEK-ZH-Nr. 2018–02357) and conducted in accordance with the principles of the Declaration of Helsinki. The present study investigated associations between DNAm of ER genes, depressive symptoms, and E2 during the transition from pregnancy to postpartum.

### Participants

Physically healthy women aged between 20 and 45 years were recruited during their third trimester of pregnancy. The participants were followed from 34 to 36 weeks of gestation up to 8–12 weeks postpartum, encompassing a mean study duration of 17 weeks per participant. Detailed information on the participants, recruitment process, and eligibility criteria can be found elsewhere [53–55]. For epigenetic analyses, the original sample size (*N* = 161) was reduced to *n* = 159, as one participant did not provide written informed consent to use (epi-)genetic data, and one participant did not provide blood samples.

### Procedure

Women interested in participating in the study were first screened for eligibility using an online questionnaire. Eligible participants were then invited to a telephone interview to confirm the inclusion and exclusion criteria. After successful inclusion, the first laboratory visit took place at around 34–36 weeks of gestation at the University of Zurich, Department of Clinical Psychology and Psychotherapy. During this visit, which started between 8 and 9 am, various psychological, biological, and (epi-)genetic parameters were assessed. Additionally, the participants were given instructions on carrying out the five home assessments, which encompassed the independent collection of saliva samples and several online questionnaires. After completion of the home assessments, the participants were invited for a second laboratory visit at approximately 8–12 weeks postpartum. During this visit, the same parameters as during the first visit were assessed, along with birth-related information.

#### Assessment of Perinatal Mood

The German version of the Edinburgh Postnatal Depression Scale (EPDS) was used to assess depressive symptoms [56, 57]. The EPDS is a validated self-report screening tool to assess depressive symptoms during pregnancy and the postpartum period, with ten items rated on a 4-point Likert scale. The original validation of the German version of the EPDS showed good internal consistency, with Cronbach’s *α* = 0.81 [56]. Participants completed the EPDS at both laboratory visits, as well as on the first day of each home assessment time point. In the present study, we used the EPDS scores from both laboratory visits.

### Blood Sampling

The participants provided up to five drops of blood (about 50 µL per drop) during the laboratory visits at 34–36 weeks of gestation and 8–12 weeks postpartum. Blood samples were collected through finger prick using the dried blood spot (DBS) method with standardized filter paper (No. 903 Whatman, DBS Protein Saver Card). The samples were dried for 3–4 h at room temperature before being stored at −20 °C at the laboratory of the University of Zurich until further biochemical analysis.

### Saliva Sampling

Saliva samples were collected using the passive drool method with SaliCap sampling tubes (IBL International GMBH, Hamburg, Germany). The participants were instructed to collect a targeted total of 52 saliva samples. Samples were collected on two consecutive days at 34–36 weeks of gestation, 40 weeks of gestation, 4–8 weeks postpartum, and 8–12 weeks postpartum. Additionally, the participants provided samples on 5 consecutive days, starting within the first 48 h after delivery. On each assessment day, four saliva samples were provided: three in the morning (immediately after awakening, 30 and 45 min after awakening) and one in the evening between 8 and 10 pm. The samples were stored in the participants’ home freezers until the second laboratory visit, whereupon they were stored at −20 °C at the laboratory of the University of Zurich until further biochemical analysis.

### Estradiol Assessment

As salivary assessment of steroid hormones is a reliable marker of serum steroid levels [58, 59], saliva samples were used to quantify E2 levels. Salivary E2 (pg/mL) was determined using luminescence immunoassay with enzyme-linked immunosorbent assay (ELISA) kits (IBL International GmbH, Hamburg, Germany, catalog number RE62141/RE62149). E2 determinations were performed by Dresden LabService GmbH in Dresden, Germany. In accordance with the manufacturer’s instructions, the standard range for E2 was between 2 and 64 pg/ml, and the highest cross-reactivity was with Estrone, at around 14%. The inter- and intraassay coefficients of variability were 9.5% and <6%, respectively. E2 values were only available for *n* = 126 participants. Missing E2 values were imputed using predictive mean matching (pmm) from the mice package in R (version 4.3.2) [60], generating 50 imputation datasets to account for the uncertainty introduced by imputing missing values. In this study, mean E2 values from the first day of the assessment at around 34–36 weeks of gestation and 8–12 weeks postpartum were used to account for inter-day variability.

### Genotyping

Genomic DNA was extracted from DBS using the MicroGEM forensicGEM Kit (MicroGEM UK, Southampton, UK). Five single nucleotide polymorphisms (SNPs) in three ER genes were genotyped using TaqMan SNP Genotyping Assays (ThermoFischer, Waltham, MA, USA): rs2234693 and rs9340799 in *ESR1*, rs1256049 and rs4986938 in *ESR2*, and rs3808350 in *GPER*. Of the 159 samples, 158 were successfully genotyped for all 5 SNPs. However, one sample failed to produce an adequate fluorescent signal to distinguish alleles for the SNP in *GPER*. Chi-square (*χ*^2^) tests were used to test whether the genotype distribution was in Hardy–Weinberg equilibrium (HWE).

### Methylation Analysis

Genomic DNA was extracted from DBS and was reported to provide reliable results in the context of methylation analysis [61]. Three punches of 3.0 mm diameter DBS were used to extract DNA with the QIAamp DNA Investigator Kit (QIAGEN, Hilden, Germany), in accordance with the manufacturer’s instructions. Additionally, duplicates and negative controls (samples without DNA) were included for quality control. The extracted DNA and negative controls were eluted in a final volume of 30μL RNase-free water. DNA concentration was assessed using NanoDrop (Thermo Fischer Scientific, Waltham, MA, USA) and ranged from 9.04 to 86.67 ng. DNA extraction was performed by a trained biologist in our laboratory at the University of Zurich, Department of Clinical Psychology, while subsequent steps were carried out at the Genetic Diversity Centre (GDC), ETH Zurich.

Genomic DNA was bisulfite-converted using the EZ-96 DNA Methylation-Lightning Kit D5032 (Zymo Research, Irvine, CA, USA) in accordance with the manufacturer’s instructions, which recommend using samples containing 0.5–2000 ng of DNA.

**Fig. 1:**
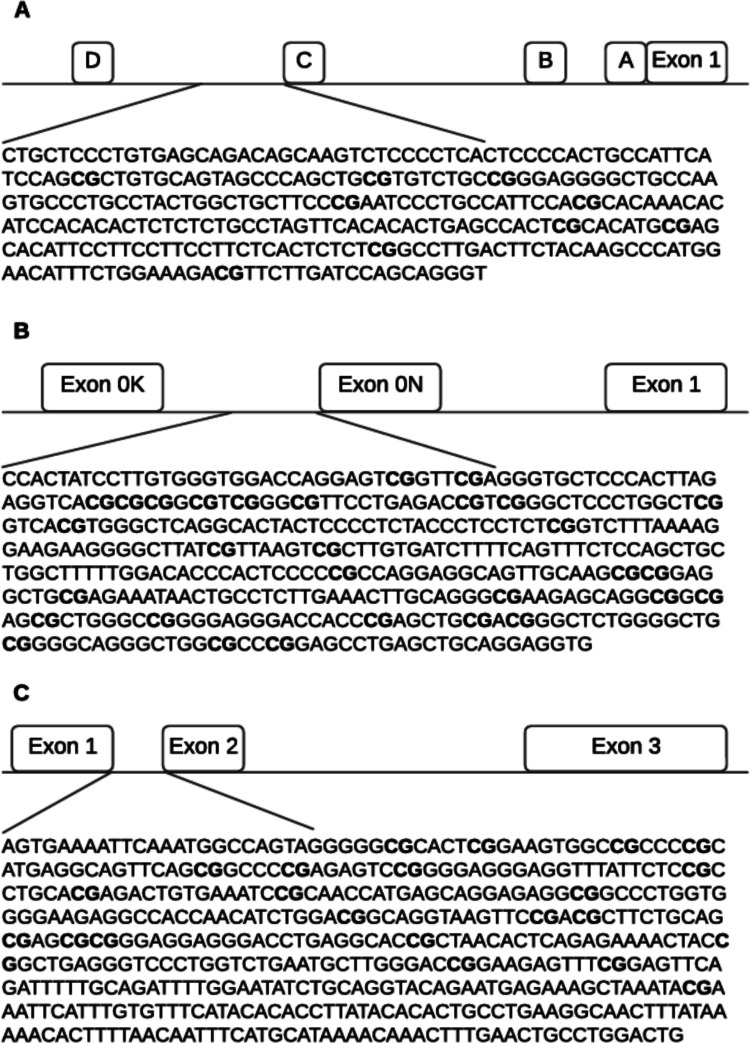
Schematic figures of *ESR1* (**a**), *ESR2* (**b**), and *GPER* (**c**) promoter regions. **a** The DNA sequence of *ESR1*, chr6: 151805523–151805822, is located in a CpG island shore of promoter C. **b** The DNA sequence of *ESR2*, chr14: 64760866–64761269, is located in a CpG island of promoter 0N. **c** The DNA sequence of *GPER*, chr7: 1087059–1087533, is located in a CpG island across exons 1 and 2. White boxes represent exons or promoter regions. Bold “CG” correspond to the interrogated CpGs

An initial polymerase chain reaction (PCR) was performed on the bisulfite-treated DNA using the Kapa HiFi Uracil+ master mix (Kapa Biosystems, Wilmington, MA, USA). Primers included the universal oligonucleotides CS1/CS2 at the 5ʹ ends (Fluidigm, San Francisco, CA, USA), which are used for customized next-generation sequencing (NGS) (see Table 1). The primers were designed to target the specific DNA sequence of the *ESR1* shore of promoter C (hg 38; chr6: 151805523–151805822, Fig. 1A), the *ESR2* promoter 0 N (hg 38; chr14: 64760866–64761269, Fig. 1B), and the *GPER* promoter (hg 38; chr7: 1087059–1087533, Fig. 1C). The PCR conditions were set to an initial temperature of 95 °C for 3 min, followed by 30× (98 °C for 20 s, annealing temperature for 15 s, and 72 °C for 15 s), and a final elongation at 72 °C for 40 s. The annealing temperature varied for each sequence (see Table 1). Subsequently, a second PCR was performed with an initial temperature of 95 °C for 3 min, then 20× (98 °C for 20 s, annealing temperature for 15 s, 72 °C for 15 s), and a final elongation at 72 °C for 40 s. After the second PCR, amplicons were purified using KingFisher (Thermo Fisher Scientific, Waltham, MA, USA).

**Table 1:** PCR primers used for amplification of DNA sequences in *ESR1*, *ESR2*, and *GPER*

Gene	Forward primer	Reverse primer	GRCh38	AT (°C)
*ESR1*	ACACTGACGACATGGTTCTACA NNN GTTTTTTGTGAGTAGATAGTAAGTT	TACGGTAGCAGAGACTTGGTCT NNN AAACCTACCCTACTAAATCAAAAAC	chr6: 151805523–151805822	55
*ESR2*	ACACTGACGACATGGTTCTACA NNN TTATTATTTTTGTGGGTGGAT	TACGGTAGCAGAGACTTGGTCT NNN CACCTCCTACAACTCAAACTC	chr14: 64760866–64761269	59
*GPER*	ACACTGACGACATGGTTCTACA NNN AGTGAAAATTTAAATGGTTAGTA	TACGGTAGCAGAGACTTGGTCT NNN ACAATCCAAACAATTCAAAATTTATTT	chr7: 1087059–1087533	57

Universal primer CS1 = ACACTGACGACATGGTTCTACA, universal primer CS2 = TACGGTAGCAGAGACTTGGTCT. Abbreviations: *PCR* polymerase chain reaction, *ESR1* estrogen receptor alpha gene, *ESR2* estrogen receptor beta gene, *GPER* G protein-coupled estrogen receptor gene, *GRCh38* Genome Reference Consortium Human Build 38 Organism, *AT* annealing temperature

Purified amplicons were then indexed with customized single barcodes (Fluidigm, San Francisco, CA, USA) using a third PCR with the conditions set to 95 °C for 3 min, then 10× (98 °C for 20 s, 58 °C for 15 s, 72 °C for 15 s), and a final elongation at 72 °C for 40 s. The indexed amplicons were eluted in 15 μL RNase-free water and again purified using KingFisher (Thermo Fisher Scientific, Waltham, MA, USA).

The purified indexed amplicons were quantified using the Sparke Plate Reader (TECAN Spark, Tecan Group Ltd, Maennedorf, Switzerland), before normalization and pooling were conducted. After a final purification with AMPure XP beads, the pool was quantified using the Agilent 2200 Tape Station instrument and HS DNA 1000 reagents (Agilent Scientific Instruments, Santa Clara, CA, USA) and Quibit (Thermo Fischer Scientific, Waltham, MA, USA). The pool was then diluted to a final molarity of 4 nM. PhiX spike-in (15%) was added to the library to increase the diversity of base calling during sequencing. The final library was sequenced on the Illumina MiSeq using the V3, 600 cycles kit (300 PE; Illumina, San Diego, CA, USA). Low-quality products were removed using Trimmomatic v0.35 (http://www.usadellab.org/cms/index.php?page=trimmomatic [62]). The remaining sequencing reads were aligned to the target regions. The Bismark program (v0.19.0) was used to extract the number of methylated (cytosine) and non-methylated (thymine) bases.

A total of 33, 18, and 12 samples in *ESR1*, *ESR2*, and *GPER*, respectively, showed zero coverage (no aligned reads detected) and were thus missing. In accordance with Chen et al. [63], a minimum threshold of 100 reads was set. For *ESR1*, *ESR2*, and *GPER*, 80, 67, and 88 samples, respectively, did not reach this threshold and were thus excluded. For the remaining samples, coverage ranged from 109 to 325′599 for *ESR1*, from 106 to 829′507 for *ESR2*, and from 111 to 292′350 for *GPER*. Moreover, samples with a significant deviation (± 3 times the interquartile range (IQR)) were excluded. For *ESR1*, *ESR2*, and *GPER*, 7, 16, and 8 samples, respectively, were below or above the IQR and thus excluded. After the exclusion of these outliers, the methylation levels reached normal distribution for all three sequences. Wilcoxon rank-sum tests were used to examine whether depressive symptoms (EPDS scores) differed between participants included and excluded. No significant differences were observed for *ESR1* at 34–36 weeks of gestation (median 4 vs. 4; *W* = 5456.5, *p* = 0.950) or 8–12 weeks postpartum (median 3 vs. 3; *W* = 9816.5, *p* = 0.864), for *ESR2* at 34–36 weeks of gestation (median 4 vs. 4; *W* = 7184, *p* = 0.814) or 8–12 weeks postpartum (median 3 vs. 3; W = 9076.5, *p* = 0.627), and for *GPER* at 34–36 weeks of gestation (median 3.5 vs. 4; *W* = 5244, *p* = 0.486) or 8–12 weeks postpartum (median 3 vs. 3; *W* = *9990.5, p* = *0.565).*

### Statistical Analysis

All analyses were performed using the overall mean DNAm levels of the 9, 30, and 22 CpGs in *ESR1*, *ESR2*, and GPER, respectively, and the DNAm levels of the individual CpGs in *ESR1*. To examine the associations between depressive symptoms, E2 levels, age, and DNAm during pregnancy and the postpartum period, we performed Spearman or Pearson correlations, depending on the normality of the data. Moreover, multivariate linear regression analyses were conducted to assess the effect of depressive symptoms and E2 levels on DNAm in pregnancy and the postpartum period, while controlling for maternal age and history of depression. In addition, to investigate potential modifying effects of ER genes on the association between depressive symptoms and DNAm, an interaction term between genetic variables and depressive symptoms was included in the multivariate linear regression models. Importantly, genotypes were analyzed as haplotypes, reconstructed by grouping SNPs within each gene using an expectation–maximization algorithm [64]. SNPs that could not be grouped by gene were analyzed individually. Additional details on haplotype reconstruction can be found in a previously published manuscript [65]. Paired *t*-tests were used to investigate DNAm changes from pregnancy to postpartum. In case of significant DNAm changes, multivariate linear regressions were calculated with the corresponding DNAm delta scores, while controlling for the previously mentioned covariates. Two sets of predictors were used: depressive symptom scores and E2 levels obtained at 34–36 weeks of gestation and delta scores of depressive symptoms and E2 levels. All statistical tests were two-sided, and the significance level was set at *p* ≤ 0.05. To correct for multiple testing, the Benjamini–Hochberg method [66] was used, with the significance threshold set at *q* ≤ 0.1. We found no issues with multicollinearity, which was assessed using the variance inflation factor (VIF; all VIFs were < 2). All statistical analyses were performed using R (version 4.3.2; R Project).

## Results

### Sample Characteristics

Sample sizes varied between variables due to the missing data. Descriptive statistics were therefore calculated for all available data points for each variable used in this study (see Table 2). Participant’s age ranged between 21 and 43 years, with a median of 33 years. Moreover, all participants (*n* = 159) were of self-reported European ancestry, with the majority reporting being Swiss (71.7%). Moreover, genotype distribution for all SNPs was consistent with the HWE (*p* > 0.05). Genotype and haplotype distribution for each corresponding DNAm region are displayed in additional file 1, Table S1-2. More information on sociodemographic characteristics can be found elsewhere [53, 55].

**Table 2:** Descriptive statistics of biological and psychological measures

Variable	34–36 weeks of gestation		8–12 weeks postpartum	
	*N*	M (SD)	*N*	M (SD)
*ESR1* CpGI shore methylation (%)	69	72.83 (5.47)	134	74.06 (5.77)
CpG 1 methylation (%)	69	61.76 (11.34)	134	62.57 (13.78)
CpG 2 methylation (%)	69	76.83 (9.35)	134	77.02 (11.05)
CpG 3 methylation (%)	69	83.20 (7.84)	134	84.35 (8.36)
CpG 4 methylation (%)	69	78.39 (8.97)	134	80.22 (9.66)
CpG 5 methylation (%)	69	81.37 (7.58)	134	81.35 (10.28)
CpG 6 methylation (%)	69	83.60 (11.95)	134	84.73 (10.15)
CpG 7 methylation (%)	69	82.84 (5.12)	134	83.16 (8.68)
CpG 8 methylation (%)	69	31.05 (5.66)	134	31.55 (6.5)
CpG 9 methylation (%)	69	76.44 (8.70)	134	80.18 (9.09)
*ESR2* promoter 0 N methylation (%)	92	0.75 (0.32)	127	0.76 (0.44)
*GPER* promoter methylation (%)	70	13.65 (3.43)	140	14.35 (4.97)
E2 (pg/mL)	126	43.00 (7.82)	126	3.66 (1.91)
EPDS	159	4.78 (4.31)	154	4.01 (4.40)

*E2* estradiol, *EPDS* Edinburgh Postnatal Depression Scale

### DNA Methylation, Depressive Symptoms, and Estradiol

#### Third Trimester of Pregnancy

Correlation results are displayed in additional file 1, Table S3-4. Spearman’s rank-order correlations revealed a weak negative association between depressive symptom scores and both overall *ESR1* DNAm (*ρ* = −0.29, *p* = 0.013, *n* = 69) and DNAm of the CpG 1 in *ESR1* (*ρ* = −0.24, *p* = 0.04, *n* = 69). Additionally, a moderate negative association emerged between depressive symptom scores and DNAm of CpG 2 in *ESR1* (*ρ* = −0.39, *p* = 0.0007, *n* = 69). Pearson’s correlations revealed a weak positive correlation between E2 levels and DNAm of CpG 5 in *ESR1* (*r* = 0.28, *p* = 0.03, *n* = 69). No further correlations were found between any of the investigated variables (all *p* > 0.05).

**Fig. 2:**
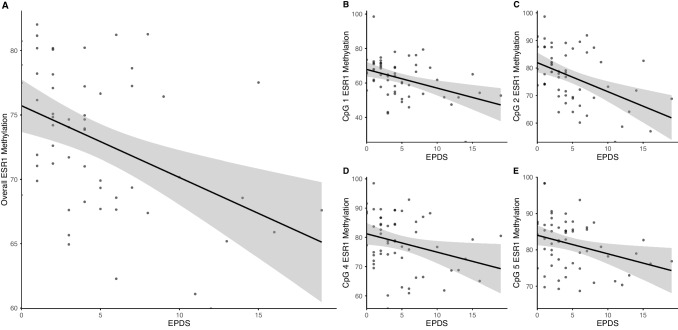
Scatterplot of EPDS scores and methylation levels of overall *ESR1* methylation and its individual CpGs. **a** Scatterplot of EPDS scores and overall *ESR1* methylation. **b** Scatterplot of EPDS scores and individual methylation of CpG 1 in *ESR1*. **c** Scatterplot of EPDS scores and individual methylation of CpG 2 in *ESR1*. **d** Scatterplot of EPDS scores and individual methylation of CpG 4 in *ESR1*. **e** Scatterplot of EPDS scores and individual methylation of CpG 5 in *ESR1*. Regression lines represent the modeled relationship with 95% confidence intervals. Abbreviations: EPDS, Edinburgh Postnatal Depression Scale

Results of the multivariate linear regressions during pregnancy are displayed in Table 3. Depressive symptoms were associated with the overall *ESR1* DNAm, as well as the DNAm of four of its individual CpGs, all of which remained significant after correction for multiple testing. In detail, increased depressive symptom scores were associated with lower DNAm levels (see Fig. 2). E2 levels were not found to be associated with the overall *ESR1*, *ESR2*, and *GPER* DNAm or with the DNAm of any of the individual CpGs in *ESR1* (all *p* > 0.05).

**Table 3:** Results of multivariate linear regressions during the third trimester of pregnancy

Methylation (%)	EPDS		E2		Age		History of depression	
	Adjusted *β*	*p*	Adjusted *β*	*p*	Adjusted *β*	*p*	Adjusted *β*	*p*
*ESR1*	−0.41	0.002*^a^	0.087	0.496	0.047	0.710	0.083	0.530
CpG 1	−0.365	0.006*^a^	0.092	0.465	0.028	0.824	0.214	0.105
CpG 2	−0.489	0.003*^a^	0.055	0.662	0.126	0.317	−0.026	0.843
CpG 3	−0.171	0.242	0.044	0.756	−0.061	0.666	0.085	0.564
CpG 4	−0.318	0.028*^a^	0.096	0.481	−0.014	0.917	−0.089	0.053
CpG 5	−0.288	0.035*^a^	0.257	0.050	0.121	0.352	0.094	0.485
CpG 6	−0.110	0.451	−0.189	0.184	0.040	0.776	0.031	0.830
CpG 7	−0.208	0.153	0.084	0.547	0.063	0.655	0.057	0.693
CpG 8	−0.255	0.071	0.217	0.109	−0.057	0.669	0.103	0.459
CpG 9	−0.222	0.129	0.073	0.601	0.003	0.981	0.047	0.745
*ESR2*	−0.026	0.833	0.165	0.166	−0.111	0.363	0.060	0.633
*GPER*	0.141	0.329	0.115	0.392	−0.214	0.117	−0.238	0.104

*p ≤ 0.05, ^a^significant after correction for multiple testing. *EPDS* Edinburgh Postnatal Depression Scale, *E2* estradiol

Additionally, exploratory follow-up analyses were conducted to compare DNAm levels between participants scoring below and above the EPDS cut-off of  ≥ 11, which has been found to maximize both sensitivity and specificity for identifying a potential diagnosis of perinatal depression [67]. Notably, only a small proportion of women scored above this threshold (7 of 69; 10.1%). Nevertheless, as shown in Table S5 and Figure S1, women with EPDS ≥ 11 exhibited significantly lower overall *ESR1* DNAm compared to those with EPDS < 11, and similar group differences were observed across the four individual CpGs.

#### Postpartum Period

Correlation results are displayed in Table S6-7. Spearman’s rank correlations revealed a weak significant positive correlation between E2 levels and both overall *GPER* DNAm (*ρ* = 0.21, *p* = 0.02, *n* = 111) and depressive symptom scores (*ρ* = 0.21, *p* = 0.01, *n* = 124). No further correlations emerged between any of the assessed variables (all *p* > 0.05). Multivariate linear regressions revealed no associations of depressive symptoms and E2 levels with the overall *ESR1*, *ESR2*, and *GPER* DNAm or with the DNAm of any of the individual CpGs in *ESR1* (all *p* > 0.05; see Table S8).

### Association Between Depressive Symptoms and DNAm, Modified by ER Genes

#### Third Trimester of Pregnancy

For *ESR1*, haplotype CA significantly modified the association between depressive symptoms and overall *ESR1* DNAm (*p* ≤ 0.001), which remained significant after correction for multiple testing. Stratified analyses among non-carriers revealed that higher depressive symptom scores were significantly associated with lower overall *ESR1* DNAm (*β* = −0.86, SE = 0.19, *p* ≤ 0.001). In contrast, among the combined group with one-copy and two-copy carriers, a trend for a positive association was found (*β* = 0.53, SE = 0.28, *p* = 0.088). No significant main or interaction effects were found for haplotype CG or TA in relation to overall *ESR1* DNAm (*p* ≥ 0.05).

Significant associations were also observed for individual CpGs in *ESR1*. Haplotype CA was found to significantly modify the association between depressive symptoms and DNAm of CpG 1 (*p* = 0.006), CpG2 (*p* = 0.004), CpG 5 (*p* = 0.01), CpG 6 (*p* ≤ 0.001), and CpG 7 (*p* ≤ 0.001), all of which remained significant after correction for multiple testing. Stratified analyses among non-carriers revealed that higher depressive symptom scores were associated with lower DNAm of CpG 1 (*β* = −1.54, SE = 0.40, *p* ≤ 0.001), CpG 2 (*β* = −1.62, SE = 0.34, *p* ≤ 0.001), CpG 5 (*β* = −0.85, SE = 0.28, *p* = 0.005), CpG 6 (*β* = −1.03, SE = 0.23, *p* ≤ 0.001), and CpG 7 (*β* = −0.68, SE = 0.20, *p* = 0.002). In contrast, among one-copy carriers, a positive association was observed for DNAm of CpG 7 (*β* = 0.80, SE = 0.34, *p* = 0.045). For haplotype CG, no significant interaction effects were observed; however, two main effects were detected. Notably, one-copy carriers showed higher DNAm of CpG 1 compared to non-carriers (*β* = 10.38, SE = 4.84, *p* = 0.03), while two-copy carriers showed higher DNAm of CpG 8 compared to non-carriers (*β* = 7.51, SE = 3.32, *p* = 0.02). Likewise, two significant main effects were observed for haplotype TA. One-copy carriers showed significantly lower DNAm of CpG 6 compared to non-carriers (*β* = −8.22, SE = 3.62, *p* = 0.028), while two-copy carriers showed significantly lower DNAm of CpG 8 compared to non-carriers (*β* = −7.72, SE = 3.45, *p* = 0.03).

For *ESR2*, haplotype groups were not found to modify the association between depressive symptoms and overall *ESR2* DNAm (*p* ≥ 0.05). However, main effects of haplotype CC and CT were observed. Specifically, two-copy carriers of haplotype CC showed lower overall *ESR2* DNAm compared to non-carriers (*β* = −0.50, SE = 0.18, *p* = 0.007). In contrast, both one-copy carriers and two-copy carriers of haplotype CT showed higher overall *ESR2* DNAm compared to non-carriers (*β* = 0.43, SE = 0.14, *p* = 0.004 and *β* = 0.48, SE = 0.19, *p* = 0.01, respectively).

For *GPER*, no significant interaction or main effects of genotype on overall *GPER* DNAm were observed (*p* > 0.05).

#### Postpartum

For *ESR1*, haplotype TA was found to significantly modify the association between depressive symptoms and overall *ESR1* DNAm (*p* = 0.036), which remained significant after correction for multiple testing. Stratified analyses indicated a trend toward a negative association among two-copy carriers (*β* = −0.97, SE = 0.47, *p* = 0.056), whereas no significant associations were observed among one-copy carriers or non-carriers (*p* > 0.05). No significant main or interaction effects were detected for the haplotypes CG or CA in relation to overall *ESR1* DNAm (*p* > 0.05).

At the site-specific level, haplotype TA also significantly modified the association between depressive symptoms and DNAm of the individual CpG 3 (*p* = 0.02), which remained significant after correction for multiple testing. Stratified analyses again revealed a trend toward a negative association among two-copy carriers (*p* > 0.05). No significant main or interaction effects were observed for haplotype CG or TA across all nine CpGs (*p* > 0.05).

For *ESR2* and *GPER*, neither main nor interaction effects were observed for the overall DNAm of *ESR2* and *GPER*, respectively (*p* > 0.05).

### DNA Methylation Changes from Pregnancy to Postpartum

**Fig. 3:**
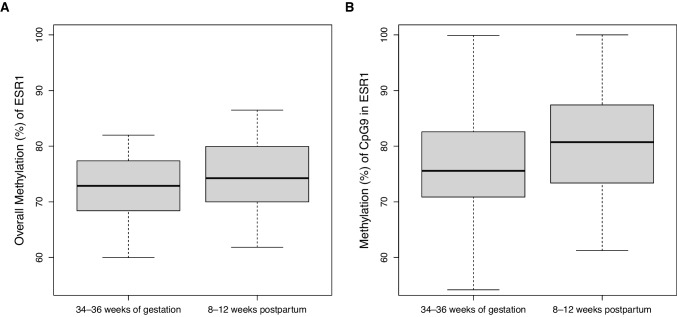
Boxplots of methylation levels at 34–36 weeks of gestation and 8–12 weeks postpartum. **a** Boxplot of overall *ESR1* methylation. **b** Boxplot of the methylation of the individual CpG 9 in *ESR1*

Results of the paired *t*-tests regarding DNAm changes in ER genes during the transition from pregnancy to postpartum are displayed in Table 4. Significant changes were found only for the overall *ESR1* DNAm and for the DNAm of the individual CpG 9 in *ESR1*, which were both found to increase (see Fig. 3). Both of these DNAm changes remained significant after correction for multiple testing.

**Table 4:** Results of the paired *t*-tests of the methylation changes from pregnancy to postpartum

Methylation (%)	*N*	34–36 weeks of gestation		8–12 weeks postpartum		*t*-statistics	*p*	Cohen’s *d*
		M	SD	M	SD			
*ESR1*	60	72.62	5.67	74.52	6.12	2.590	0.012*^a^	0.334
CpG 1	60	61.48	11.31	62.24	16.93	0.311	0.756	0.040
CpG 2	60	76.10	9.39	76.82	12.74	0.430	0.668	0.055
CpG 3	60	83.66	7.08	84.94	9.18	0.918	0.361	0.118
CpG 4	60	78.08	9.46	79.73	9.53	1.041	0.302	0.134
CpG 5	60	81.04	7.87	81.19	11.78	0.096	0.923	0.012
CpG 6	60	83.24	12.73	84.85	7.46	0.962	0.339	0.124
CpG 7	60	82.76	5.35	83.21	10.81	0.339	0.735	0.043
CpG 8	60	31.31	5.60	31.49	7.49	0.156	0.876	0.020
CpG 9	60	75.87	9.12	80.79	9.57	3.151	0.002*^a^	0.406
*ESR2*	71	0.79	0.31	0.79	0.42	0.012	0.990	0.001
*GPER*	62	13.72	3.6	14.65	5.3	1.095	0.277	0.139

*p ≤ *0.05, *^a^significant after correction for multiple testing

### Change Scores from Pregnancy to Postpartum

**Fig. 4:**
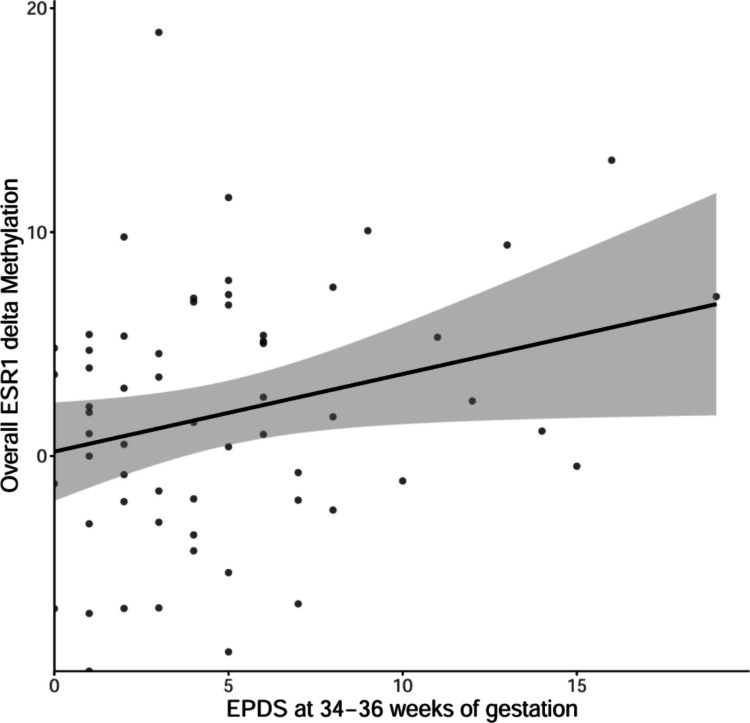
Scatterplot of depressive symptom scores at 34–36 weeks of gestation and overall *ESR1* delta methylation. Regression lines represent the modeled relationship with 95% confidence intervals. Abbreviations: EPDS, Edinburgh Postnatal Depression Scale

Change scores (delta values) were only calculated for the entire *ESR1* DNAm and for the DNAm of the individual CpG 9 in *ESR1*, as only these changed significantly during the transition from pregnancy to postpartum. Multivariate linear regression analysis did not reveal any significant associations when using delta scores of the predictors from 34–36 weeks of gestation to 8–12 weeks postpartum (all *p* > 0.05, see Table S9). An exploratory follow-up analysis using delta E2 scores from the third trimester to the first 48 h postpartum also yielded no significant results (all *p* > 0.05). However, a further exploratory analysis using scores at 34–36 weeks of gestation as the predictors revealed a significant association for delta scores of overall *ESR1* DNAm (see Table S10). Specifically, depressive symptoms at 34–36 weeks of gestation showed a significant positive association with overall *ESR1* delta DNAm (*β* = 0.311, *p* = 0.036, see Fig. 4). This finding remained significant after correction for multiple testing.

## Discussion

This longitudinal study investigated the association between DNAm of ER genes (*ESR1*, *ESR2*, and *GPER*), E2, and depressive symptoms during the transition from pregnancy to the postpartum period. Mean DNAm levels varied across the genes analyzed, with *ESR1* showing intermediate methylation, *ESR2* almost absent methylation, and *GPER* low methylation during both pregnancy and the postpartum period. During pregnancy, depressive symptoms were found to be negatively associated with the overall DNAm of the CpGI shore of *ESR1* and with four of its individual CpGs (CpG 1, CpG 2, CpG 4, and CpG 5). During the postpartum period, no associations were identified between depressive symptoms and DNAm of the three genes analyzed. Likewise, E2 was not found to be associated with DNAm of any of the three genes at either time point. In addition, the overall DNAm of the CpGI shore of *ESR1* and its individual CpG 9 increased from pregnancy to the postpartum period. Although changes in E2 levels and depressive symptoms were not linked to these DNAm changes, higher depressive symptoms at 34–36 weeks of gestation were associated with a larger increase in the overall DNAm of the CpGI shore of *ESR1*.

Recently, a systematic review by our research group described that sensitivity to estrogen signaling, in particular via ER-α, is associated with perinatal depression [68]. Markers of this sensitivity can help to identify women at risk. In this vein, the present study revealed that DNAm of the CpGI shore of *ESR1*, encoding for ER-α, was associated with depressive symptoms during pregnancy, whereas no such association was found for *ESR2* and *GPER*. These results support the assumption from other genetic studies that ER-α plays a more important role in mood regulation than do ER-β and GPER, which might be explained by its predominant expression in neuronal areas implicated in emotional functions, such as the amygdala and hypothalamus, compared to other ERs [69, 70]. Interestingly, lower DNAm levels of the overall CpGI shore of *ESR1* and its individual CpG 1, CpG 2, CpG 4, and CpG 5 were associated with increased depressive symptom scores during pregnancy. These findings were further supported by exploratory follow-up comparisons, which indicated that women scoring above the proposed EPDS cut-off of ≥ 11 exhibited lower overall *ESR1* DNAm as well as reduced DNAm across the four individual CpGs compared to those below the cut-off. While these results suggest that clinically significant depressive symptoms may be linked to altered *ESR1* DNAm patterns, they should be interpreted with caution, as only a small proportion of participants exceeded the cut-off, limiting statistical power and the generalizability of the findings. Thus, replication in larger samples enriched for clinically significant levels of perinatal depressive symptoms will be essential to clarify the robustness of these associations. Notably, higher DNAm levels of the CpGI shore of *ESR1* have been associated with lower mRNA expression of ER-α [71]. Moreover, DNAm of one of these individual CpGs, namely CpG 4, was previously found to be negatively correlated with ER-α expression [72]. Therefore, we suggest that lower DNAm levels of the CpGI shore of *ESR1*, particularly at CpG4, may upregulate ER-α expression, thereby increasing sensitivity to estrogen and thus susceptibility to depressive symptoms during pregnancy, when estrogen levels are high.

Moreover, potential modifying effects of ER gene variations on the association between perinatal depressive symptoms and DNAm of ER genes were investigated. The results indicate that such moderating effects are specific to *ESR1*, while *ESR2* haplotypes were primarily associated with main effects on DNAm, and no significant associations emerged for *GPER*. During pregnancy, haplotype CA significantly moderated the associations between perinatal depressive symptoms and both overall DNAm of *ESR1* and several individual CpGs in *ESR1*. Notably, non-carriers of haplotype CA showed robust negative associations between perinatal depressive symptoms and *ESR1* DNAm (both overall and individual CpGs 1, 2, 5, 6, and 7), consistent with the notion that higher symptom levels relate to reduced DNAm. In contrast, carriers of haplotype CA displayed attenuated or even opposite associations, suggesting that this haplotype alters the directionality of the depression–methylation relationship. These findings support the idea of genetic moderation, in which specific haplotypes shape the sensitivity of epigenetic states to psychological exposures. However, it is important to note that the group of two-copy carriers of haplotype CA was very small (*n* = 3) and was therefore combined with one-copy carriers, which may have obscured potential dose-dependent effects. In addition to these interaction effects, we also observed main genetic effects on DNAm. Specifically, among two-copy carriers, haplotype CC in *ESR2* was found to be associated with lower overall *ESR2* DNAm, whereas haplotype CT in *ESR2* was associated with higher overall *ESR2* DNAm in both one- and two-copy carriers compared to non-carriers. These patterns suggest stable, genotype-driven differences in overall *ESR2* DNAm, independent of depressive symptom levels. Taken together, these findings suggest that different ER gene variants may contribute to DNAm regulation through distinct mechanisms: some acting as stable genetic determinants, others functioning as moderators of environmentally linked epigenetic variation. Importantly, the findings underscore the value of integrative models that incorporate genetic, epigenetic, and environmental factors, rather than considering factors in isolation. However, the limited sample size within some haplotype groups requires cautious interpretation and highlights the need for replication in larger samples.

Previous cross-sectional findings by our research group indicated that mean DNAm levels of the CpGI shore of *ESR1* are associated with E2 levels within different menopausal groups [51]. In detail, E2 was positively associated with ESR1 DNAm in pre- and postmenopausal women, while a negative association was found in perimenopausal women. Contrary to our expectation, in the present study, no associations were observed between E2 levels and DNAm levels of any ER gene in either pregnant or postpartum women. Nevertheless, a trend emerged for the DNAm of CpG 5 of the CpGI shore of *ESR1*, showing a positive association with E2 in pregnant women and a negative association in postpartum women. Additionally, a trend towards a positive association between E2 and *GPER* DNAm was observed in postpartum women. These findings may reflect a subtle but dynamic and context-dependent hormonal regulation of ER gene DNAm during the perinatal period. The lack of robust associations may be due to the specific period investigated, which encompasses pronounced hormonal fluctuations. Therefore, single time point measurements may not capture the dynamic interplay between fluctuating hormone levels and DNAm, limiting our ability to detect potential long-term exposures relevant to DNAm.

In contrast to genetic variations, DNAm is an epigenetic modification that can change over time, a dynamic that has also been observed for various CpGs during the perinatal period [48, 49]. Supporting this, the present longitudinal analysis revealed that overall DNAm of the CpGI shore of *ESR1* and its individual CpG 9 increased from pregnancy to the postpartum period. Interestingly, DNAm of the CpG 9 has also been found to differ cross-sectionally between pre- and postmenopausal women [51]. As such, our findings support the assumption that DNAm patterns of ER genes, particularly at specific CpGs, may change over time and across reproductive transitions. Nevertheless, it is important to note that the increase in DNAm only showed a small effect size and that we did not assess gene expression levels to investigate corresponding changes at the receptor level. Moreover, the factors implicated in these changes still remain largely unclear. Emerging evidence from Guintivano et al. suggests that DNAm marks associated with a risk of postpartum depression overlap with estradiol-induced methylation changes [25]. Extending this finding, Mehta et al. employed a hormonal manipulation model using the gonadotropin-releasing hormone agonist (GnRHa) to examine hormone-induced mood changes in relation to a previously identified set of 116 genes enriched for estrogen receptor targets and associated with perinatal depression [73]. Notably, in women exposed to GnRHa, changes in DNAm over the course of the intervention were associated with changes in both estrogen levels and depressive symptoms. In the present study, contrary to expectation, we did not find an association of DNAm changes of ER genes with change scores either of E2 or depressive symptoms. However, when using depression scores at 34–36 weeks of gestation as predictors, higher depressive symptom scores were associated with a higher increase in *ESR1* DNAm, potentially indicating sensitivity to epigenetic changes during the transition from pregnancy to postpartum in vulnerable women with elevated depressive symptoms during pregnancy. Importantly, given the exploratory nature of this finding and the limited sample size, it should be considered preliminary and interpreted with caution, warranting replication in larger cohorts.

This is the first study to investigate DNAm levels of all three estrogen receptor genes and their association with E2 and depressive symptoms during the perinatal period. A main strength of our study lies in its longitudinal design, which, given the dynamic nature of DNAm, allowed us to examine the stability and change of ER gene methylation during the perinatal period. In view of the initial evidence from EWAS highlighting the role of estrogen signaling pathways in perinatal depression, the use of a targeted candidate gene approach overcomes the limitation of multiple testing correction [44]. A further strength of this study is the use of DBS, which is a minimally invasive method of blood sampling [74, 75] and has been found to provide high-quality methylation results that are highly correlated with more invasive methods such as venous blood samples [76].

However, some limitations of the study should also be mentioned. The correlational nature of the study precludes any conclusions about the effects of underlying pathways such as gene expression levels. For instance, as we did not assess ER gene expression levels, it remains unclear whether DNAm levels and changes are associated with corresponding gene expression levels and changes. Moreover, due to the lack of assessment of cell type-specific methylation, it cannot be ruled out that the observed changes in methylation levels reflect differences in cell type composition rather than epigenetic variability [77]. Additionally, DNAm was only assessed in peripheral blood, which may not fully capture tissue-specific epigenetic patterns relevant to brain function, given the tissue specificity of DNAm. However, peripheral DNAm has been proposed as a promising target for identifying clinically relevant biomarkers [78]. A further limitation is that we considered only a limited number of covariates. Factors known to influence DNA methylation, such as stress exposure and nutrition [79, 80], were not assessed and may have contributed to variability in DNAm patterns. Future studies should include a broader range of relevant covariates to better capture environmental and lifestyle influences on DNAm. Moreover, the validity and sensitivity of salivary E2 assessment should be acknowledged as a limitation. As was highlighted in a recent review, salivary concentrations of sex steroid hormones are substantially lower than those found in serum or plasma, which may have contributed to the null findings regarding E2 [81]. In addition, the ELISA method, like other immunoassays, is susceptible to low sensitivity due to potential cross-reactivity compared to liquid chromatography-mass spectrometry (LC–MS/MS) [82]. Furthermore, it is important to note that the expected salivary E2 range in the third trimester is approximately 24–75 pg/mL, which overlaps with the upper sensitivity threshold of the ELISA (> 64 pg/mL) used in this study [81]. This overlap led to nearly half of all missing E2 values being missing due to exceeding the upper threshold, potentially introducing bias. Finally, although this study employed a longitudinal design, it only covered a limited period within the peripartum and did not include assessments of methylation prior to pregnancy. Consequently, we cannot infer changes across the entire perinatal period, and the relatively short window of observation may have been insufficient to capture long-term epigenetic changes or cumulative effects over time.

In conclusion, the present findings indicate that lower DNAm levels of the CpGI shore of *ESR1*, which may reflect higher ER-α expression and thus greater sensitivity to estrogen, are associated with increased depressive symptoms during pregnancy. Moreover, the results add to previous findings of perinatal DNAm changes by demonstrating that ER gene methylation changes during the transition from pregnancy to the postpartum period. Although current evidence is insufficient to establish these epigenetic signatures as biomarkers of perinatal depression, the findings highlight molecular pathways of estrogen sensitivity as a promising target for future research. Furthermore, our results emphasize the need to take into account the temporal variability of DNAm patterns in order to identify reliable biomarkers. Longitudinal studies including assessments prior to pregnancy and the investigation of corresponding gene expression dynamics are warranted. Addressing these research gaps is essential to improve our understanding and early detection of perinatal mood disorders.

## Supplementary Information

Below is the link to the electronic supplementary material.

ESM 1 (DOCX 57.3 KB)

## Data Availability

The datasets generated during and/or analyzed during the current study are not publicly available as patient-specific data could be used to identify patients with great effort, but are available from the corresponding author on reasonable request.

## Abbreviations

CpG: Cytosine-guanine dinucleotide
CpGI: Cytosine-guanine dinucleotide island
DBS: Dried blood spots
DNAm: DNA methylation
ELISA: Enzyme-linked immunosorbent assay
ER: Estrogen receptor
ER-α: Estrogen receptor alpha
ER-β: Estrogen receptor beta
*ESR1*: Estrogen receptor gene 1
*ESR2*: Estrogen receptor gene 2
EPDS: Edinburgh Postnatal Depression Scale
E2: Estradiol
EWAS: Epigenome wide association studies
GABA: Gamma-aminobutyric acid
GPER: G protein-coupled estrogen receptor
HWE: Hardy-Weinberg equilibrium
NGS: Next-generation sequencing
PCR: Polymerase chain reaction
pmm: Predictive mean matching
VIF: Variance inflation factor
