# Supplementary material for: Longitudinal Analysis of Estrogen Receptor Gene Methylation, Estradiol, and Depressive Symptoms During the Perinatal Period
Source: Mol Neurobiol. 2025 Dec 1;63(1):234. doi: 10.1007/s12035-025-05556-3 (PMC12669334; doi:10.1007/s12035-025-05556-3)
Supplement: Supplementary file 1 — (DOCX 57.3 KB) [file 12035_2025_5556_MOESM1_ESM.docx]

**Supplementary material for:**

**Longitudinal Analysis of Estrogen Receptor Gene Methylation, Estradiol, and Depressive Symptoms during the Perinatal Period**

Gianna Zorzini, MSc^a^, Alexandra Johann, PhD^a^, Jelena Dukic, MSc^a^, Elena Gardini, PhD^a^ & Ulrike Ehlert, PhD^a^

^a^ Department of Clinical Psychology and Psychotherapy, University of Zurich, Zurich, Switzerland

**Corresponding author:**

Prof. Dr. Ulrike Ehlert, University of Zurich, Institute of Psychology, Clinical Psychology and Psychotherapy, Binzmuehlestrasse 14 / Box 26, 8050 Zurich, Switzerland, Telephone: +41 44 635 73 50, E-mail: u.ehlert@psychologie.uzh.ch

**Contents**

Table S1. Absolute frequencies and percentages (in brackets) of reconstructed haplotypes for *ESR1* and *ESR2* methylation regions………………………………………………………………………………………………...2

Table S2. Absolute frequencies and percentages of the genotype distribution for GPER methylation region…….2

Table S3. Pearson correlations of overall *ESR1*, *ESR2* and *GPER* methylation at 34-36 weeks of gestation………..2

Table S4. Pearson correlations of individual CpG sites in *ESR1* at 34-36 weeks of gestation………………….……2

Table S5. Exploratory follow-up comparisons of overall *ESR1* DNAm and four of its individual CpGs by EPDS cut-off (<11 vs. ≥11)………………………………………………………………………………………………...3

Table S6. Pearson correlations of overall *ESR1*, *ESR2* and *GPER* methylation at 8-12 weeks postpartum….……3

Table S7. Spearman correlations of individual CpG sites in *ESR1* at 8-12 weeks postpartum…………………….3

Table S8. Results of multivariate linear regression during the postpartum period…………………………………3

Table S9. Results of multivariate linear regression predicting delta methylation based on change scores…….…..4

Table S10. Results of multivariate linear regression predicting delta methylation based on scores from pregnancy…………………………………………………………………………………………………………..4

Figure S1. Boxplots of methylation levels at 34-36 weeks of gestation by EPDS cut-off groups (<11 vs. ≥11)….4

| **Table S1**  Absolute frequencies and percentages (in brackets) of reconstructed haplotypes for *ESR1* and *ESR2* methylation regions. | | | | | | | | |
| --- | --- | --- | --- | --- | --- | --- | --- | --- |
| Methylation region | SNPs | Haplotype | Haplotype frequency at 34.-36. weeks of gestation | | | Haplotype frequency at 8-12 weeks postpartum | | |
|  |  |  | 0 | 1 | 2 | 0 | 1 | 2 |
| ESR1 | rs2234693, rs9340799 | CG  TA  CA | 28 (40.6%)  17 (24.6%)  55 (79.7%) | 31 (44.9%)  33 (47.8%)  14 (20.3%) | 10 (14.5%)  19 (27.6%)  0 (0%) | 50 (37.3%)  30 (22.3%)  106 (79.1%) | 67 (50.0%)  72 (53.8%)  28 (20.9%) | 17 (12.7%)  32 (23.9%)  0 (0%) |
| ESR2 | rs1256049, rs4986938 | CC  CT  TC  TT | 13 (14.1%)  44 (47.8%)  82 (89.1%)  92 (100%) | 42 (45.7%)  38 (41.3%)  10 (10.9%)  0 (0%) | 37 (40.1%)  10 (10.9%)  0 (0%)  0 (0%) | 19 (14.9%)  59 (46.5%)  112 (88.2%)  127 (0.0%) | 61 (48.1%)  53 (41.7%)  15 (11.8%)  0 (0%) | 47 (37.0%)  15 (11.8%)  0 (0%)  0(0%) |
|  | | | | | |  |  |  |

| **Table S2**  Absolute frequencies and percentages of the genotype distribution for GPER methylation region. | | | | | | | | |
| --- | --- | --- | --- | --- | --- | --- | --- | --- |
| Methylation region | SNP | Genetic Variation | Genotype 34.-36 weeks of gestation | | | Genotype 8-12 weeks postpartum | | |
|  |  |  | AA | AG | GG | AA | AG | GG |
| GPER | rs3808350 | A>G | 28 (40.5%) | 30 (43.5%) | 11 (16.0%) | 63 (45.3%) | 59 (42.4%) | 17 (12.2%) |
| Note. SNP Single Nucleotide Polymorphism. | | | | | | | | |

| **Table S3**  Pearson correlations of overall *ESR1*, *ESR2* and *GPER* methylation at 34-36 weeks of gestation. | | | | | | | |
| --- | --- | --- | --- | --- | --- | --- | --- |
| **Variable** | **N** | **1** | **2** | **3** | **4** | **5** | **6** |
| 1. *ESR1* Methylation | 69 | - |  |  |  |  |  |
| 2. *ESR2* Methylation | 92 | .14 | - |  |  |  |  |
| 3. *GPER* Methylation | 70 | -.23 | .14 | - |  |  |  |
| 4. EPDS | 159 | -.29^a^* | -.01^a^ | .15^a^ | - |  |  |
| 5. E2 | 126 | .10 | .17 | .12 | .05^a^ | - |  |
| 6. Age | 159 | .09 | -.11 | -.23 | .004^a^ | .007 | - |
| Note. **p* < .05, ^a^Spearman rank correlation, EPDS Edinburgh Postnatal Depression Scale, E2 estradiol. | | | | | | | |

| **Table S4**  Pearson correlations of individual CpG sites in *ESR1* at 34-36 weeks of gestation. | | | | | | | | | | | | | |
| --- | --- | --- | --- | --- | --- | --- | --- | --- | --- | --- | --- | --- | --- |
| **Variable** | **N** | **1** | **2** | **3** | **4** | **5** | **6** | **7** | **8** | **9** | **10** | **11** | **12** |
| 1. CpG 1 | 69 | - |  |  |  |  |  |  |  |  |  |  |  |
| 2. CpG 2 | 69 | .71* | - |  |  |  |  |  |  |  |  |  |  |
| 3. CpG 3 | 69 | .52^a^* | .53^a^* | - |  |  |  |  |  |  |  |  |  |
| 4. CpG 4 | 69 | .65* | .76* | .33^a^* | - |  |  |  |  |  |  |  |  |
| 5. CpG 5 | 69 | .65* | .64* | .34^a^* | .61* | - |  |  |  |  |  |  |  |
| 6. CpG 6 | 69 | .59^a^* | .51^a^* | .24^a^* | .47^a^* | .52^a^* | - |  |  |  |  |  |  |
| 7. CpG 7 | 69 | .51* | .61* | .31^a^* | .52* | .71* | .38^a^* | - |  |  |  |  |  |
| 8. CpG 8 | 69 | .29^a^* | .28^a^* | .17^a^ | .29^a^* | .51^a^* | .11^a^ | .28^a^* | - |  |  |  |  |
| 9. CpG 9 | 69 | .44* | .59* | .41^a^* | .44* | .65* | .45^a^* | .56* | .47^a^* | - |  |  |  |
| 10. EPDS | 159 | -.24^a^* | -.39^a^* | -.21^a^ | -.19^a^ | -.14^a^ | -.18^a^ | -.17^a^ | -.11^a^ | -.13^a^ | - |  |  |
| 11. E2 | 126 | .08 | .08 | .08^a^ | .09 | .28* | -.12^a^ | .11 | .15^a^ | .08 | .05^a^ | - |  |
| 12. Age | 159 | .12 | .12 | .11^a^ | .04 | .12 | -.05^a^ | -.01 | .07^a^ | -.001 | .004^a^ | .007 | - |
| Note. **p* < .05, ^a^Spearman rank correlation, EPDS Edinburgh Postnatal Depression Scale, E2 estradiol. | | | | | | | | | | | | | |

| **Table S5**  Exploratory follow-up comparisons of overall *ESR1* DNAm and four of its individual CpGs by EPDS cut-off (<11 vs. ≥11). | | | | |
| --- | --- | --- | --- | --- |
| Methylation (%) | EPDS < 11  (n=62) | EPDS ≥ 11  (n=7) | *W* | *p* |
|  | M(SD) | M(SD) |  |  |
| *ESR1* | 73.5(5.0) | 66.5(5.8) | 362 | 0.004 |
| CpG 1 | 63.1(10.5) | 49.7(11.9) | 352 | 0.007 |
| CpG 2 | 78.2(8.4) | 65.1(10.1) | 365 | 0.003 |
| CpG 4 | 79.2(8.8) | 71.0(6.9) | 336 | 0.018 |
| CpG 5 | 82.0(7.6) | 75.6(4.4) | 334 | 0.021 |
| Note. Group differences were tested using Wilcoxon rank-sum tests. EPDS Edinburgh Postnatal Depression Scale. | | | | |

| **Table S6**  Pearson correlations of overall *ESR1*, *ESR2* and *GPER* methylation at 8-12 weeks postpartum. | | | | | | | |
| --- | --- | --- | --- | --- | --- | --- | --- |
| **Variable** | **N** | **1** | **2** | **3** | **4** | **5** | **6** |
| 1. *ESR1* Methylation | 134 | - |  |  |  |  |  |
| 2. *ESR2* Methylation | 127 | -.11^a^ | - |  |  |  |  |
| 3. *GPER* Methylation | 135 | -.03 | .07^a^ | - |  |  |  |
| 4. EPDS | 140 | -.01^a^ | .04^a^ | .006^a^ | - |  |  |
| 5. E2 | 126 | -.08^a^ | -.04^a^ | .21^a^* | .21^a^* | - |  |
| 6. Age | 159 | -.07 | .13^a^ | .01 | -.01^a^ | .12^a^ | - |
| Note. **p* < .05, ^a^Spearman rank correlation, EPDS Edinburgh Postnatal Depression Scale, E2 estradiol. | | | | | | | |

| **Table S7**  Spearman correlations of individual CpG sites in *ESR1* at 8-12 weeks postpartum. | | | | | | | | | | | | | |
| --- | --- | --- | --- | --- | --- | --- | --- | --- | --- | --- | --- | --- | --- |
| **Variable** | **N** | **1** | **2** | **3** | **4** | **5** | **6** | **7** | **8** | **9** | **10** | **11** | **12** |
| 1. CpG1 | 134 | - |  |  |  |  |  |  |  |  |  |  |  |
| 2. CpG2 | 134 | .39* | - |  |  |  |  |  |  |  |  |  |  |
| 3. CpG 3 | 134 | .46* | .61* | - |  |  |  |  |  |  |  |  |  |
| 4. CpG 4 | 134 | .33* | .54* | .51^a^* | - |  |  |  |  |  |  |  |  |
| 5. CpG5 | 134 | .32* | .49* | .54* | .59* | - |  |  |  |  |  |  |  |
| 6. CpG 6 | 134 | .29* | .43* | .34* | .46* | .43* | - |  |  |  |  |  |  |
| 7. CpG 7 | 134 | .35* | .57* | .42* | .55* | .47* | .54* | - |  |  |  |  |  |
| 8. CpG 8 | 134 | .26* | .35* | .36* | .36* | .32* | .25* | .41* | - |  |  |  |  |
| 9. CpG9 | 134 | .22* | .41* | .19^a^* | .49^a^* | .46* | .26* | .43* | .33* | - |  |  |  |
| 10. EPDS | 154 | -.01 | .01 | -.002 | -.01 | -.02 | -.01 | -.08 | -.05 | -.12 | - |  |  |
| 11. E2 | 126 | -.06 | -.19* | -.15 | -.03 | -.09 | -.01 | -.04 | -.01 | -.05 | .21* | - |  |
| 12. Age | 159 | -.05 | -.008 | -.06^a^ | -.09^a^ | .09 | -.06 | -.04 | -.01 | -.04^a^ | -.01 | .12 | - |
| Note. **p* < .05, ^a^Pearson correlation, EPDS Edinburgh Postnatal Depression Scale, E2 estradiol. | | | | | | | | | | | | | |

| **Table S8**  Results of multivariate linear regression during the postpartum period. | | | | | | | | |
| --- | --- | --- | --- | --- | --- | --- | --- | --- |
| **Variable** | **EPDS** |  | **E2** |  | **Age** |  | **History of depression** | |
|  | **β** | ***p*** | **β** | ***p*** | **β** | ***p*** | **β** | ***p*** |
| *ESR1* | 0.055 | 0.590 | -0.153 | 0.130 | -0.040 | 0.690 | 0.133 | 0.193 |
| CpG 1 | 0.030 | 0.768 | -0.077 | 0.45 | 0.012 | 0.904 | -0.030 | 0.766 |
| CpG 2 | 0.056 | 0.568 | -0.130 | 0.187 | -0.066 | 0.495 | 0.275 | 0.006 |
| CpG 3 | 0.038 | 0.707 | -0.114 | 0.262 | -0.048 | 0.635 | 0.070 | 0.491 |
| CpG 4 | -0.074 | 0.473 | -0.046 | 0.652 | -0.022 | 0.829 | -0.035 | 0.731 |
| CpG 5 | 0.009 | 0.929 | -0.188 | 0.064 | 0.037 | 0.707 | 0.035 | 0.730 |
| CpG 6 | 0.081 | 0.432 | -0.061 | 0.549 | -0.022 | 0.825 | -0.013 | 0.898 |
| CpG 7 | 0.071 | 0.491 | -0.039 | 0.696 | -0.061 | 0.542 | 0.160 | 0.119 |
| CpG 8 | 0.075 | 0.467 | -0.088 | 0.390 | 0.010 | 0.915 | 0.040 | 0.698 |
| CpG 9 | -0.184 | 0.073 | -0.046 | 0.642 | -0.033 | 0.740 | 0.036 | 0.649 |
| *ESR2* | -0.010 | 0.924 | 0.001 | 0.989 | 0.057 | 0.584 | 0.137 | 0.194 |
| *GPER* | -0.001 | 0.992 | 0.185 | 0.059 | 0.0432 | 0.662 | 0.086 | 0.386 |
| Note. EPDS Edinburgh Postnatal Depression Scale, E2 estradiol. | | | | | | | | |

| **Table S9**  Results of multivariate linear regression predicting delta methylation based on change scores. | | | | | | | | |
| --- | --- | --- | --- | --- | --- | --- | --- | --- |
| **Variable** | **Delta EPDS** | | **Delta E2** | | **Age** | | **History of depression** | |
|  | β | *p* | β | *p* | β | *p* | β | *p* |
| Delta *ESR1* | -0.070 | 0.643 | -0.187 | 0.209 | -0.163 | 0.281 | -0.072 | 0.628 |
| Delta CpG 9 | -0.039 | 0.800 | 0.081 | 0.587 | -0.081 | 0.596 | -0.121 | 0.428 |
| Note. EPDS Edinburgh Postnatal Depression Scale, E2 estradiol. | | | | | | | | |
|  |  |  |  |  |  |  |  |  |

| **Table S10**  Results of multivariate linear regression predicting delta methylation based on scores from pregnancy. | | | | | | | | |
| --- | --- | --- | --- | --- | --- | --- | --- | --- |
| **Variable** | **EPDS at 34-36 weeks of gestation** | | **E2 at 34-36 weeks of gestation** | | **Age** | | **History of depression** | |
|  | β | *p* | β | *p* | β | *p* | β | *p* |
| Delta *ESR1* | 0.311 | 0.036*^a^ | 0.216 | 0.121 | -0.248 | 0.078 | 0.032 | 0.823 |
| Delta CpG 9 | -0.091 | 0.560 | -0.098 | 0.511 | -0.013 | 0.927 | -0.178 | 0.260 |
| Note. **p* < .05, ^a^significant after correction for multiple testing, EPDS Edinburgh Postnatal Depression Scale, E2 estradiol. | | | | | | | | |
|  |  |  |  |  |  |  |  |  |


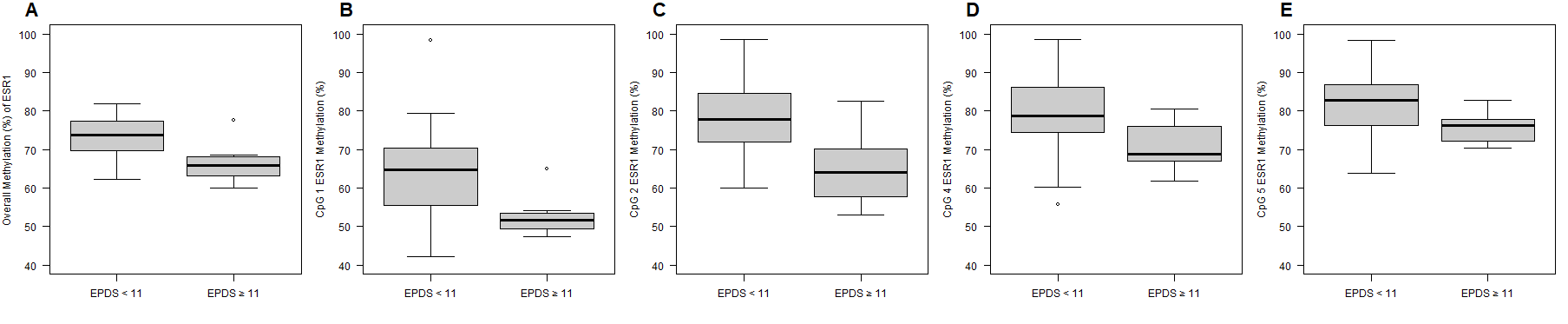


**Fig. S1** Boxplots of methylation levels at 34-36 weeks of gestation by EPDS cut-off groups (<11 vs. ≥11). **a.** Boxplot of overall *ESR1* methylation. **b.** Boxplot of methylation of CpG 1 in *ESR1*. **c.** Boxplot methylation of CpG 2 in *ESR1*. **d.** Boxplot of methylation of CpG 4 in *ESR1*. **e.** Boxplot of methylation of CpG 5 in *ESR1*. Abbreviations: EPDS Edinburgh Postnatal Depression Scale
